# CT Radiomics–Based Machine Learning Model for Predicting Capsular and Neural Invasion in Thyroid Carcinoma: Diagnostic Accuracy Study

**DOI:** 10.2196/77349

**Published:** 2026-03-12

**Authors:** Fang-fang Cong, Ke Tian, Qian Gao, Fulin Wang, Peng Sun, Nan Xu

**Affiliations:** 1 Department of MRI, Zhoukou Medical Science Research Center, Zhoukou Central Hospital, Zhoukou, China; 2 Department of Radiology, Air Force Medical Center, Air Force Medical University, Number 30 Fucheng Road, Haidian District, Beijing, 100142, China, 86 18510230072; 3 Department of CT, Zhoukou Medical Science Research Center, Zhoukou Central Hospital, Zhoukou, China

**Keywords:** thyroid carcinoma, capsular invasion, neural invasion, tadiomics, computed tomography, CT, artificial intelligence, machine learning

## Abstract

**Background:**

Thyroid carcinoma is the most prevalent endocrine malignancy, with a worldwide increasing incidence. Capsular invasion and neural invasion (NI) are pivotal prognostic factors for recurrence and survival; however, their preoperative noninvasive assessment remains challenging.

**Objective:**

We aimed to identify computed tomography (CT) radiomic biomarkers associated with capsular invasion in thyroid carcinoma, construct machine learning models integrating radiomic and clinical data, and evaluate their utility for NI risk stratification.

**Methods:**

In this retrospective cohort, 111 patients with thyroid carcinoma were divided into capsular invasion–positive (n=63) and capsular invasion–negative (n=48) groups, with 37 (33.3%) cases presenting concurrent NI. Radiomic features were extracted from arterial and venous phase CT images at original resolution, including 111 gray-level co-occurrence matrix features. Nine key radiomic features (A1-A9) were selected via least absolute shrinkage and selection operator regression (λ=0.017). To preserve the physical meaning of texture features (eg, spatial correlation and contrast reflecting tumor microstructural heterogeneity), no resampling or scaling was performed on the regions of interest during radiomic feature extraction. Nomogram models and random forest (RF) models were constructed based on clinical indicators (galectin-3, etc) and radiomic features, respectively. Additionally, a neural network (NN) model integrating multimodal data was developed. Model stability was verified using 5-fold cross-validation and 1000-time bootstrap resampling, while performance was evaluated via receiver operating characteristic curves, calibration curves, and decision curve analysis.

**Results:**

Model performance analysis revealed that among the nomogram models, the clinical indicator-based nomogram achieved an internally estimated area under the curve (AUC) of 0.9418 (95% CI 0.892‐0.976) in the capsular invasion prediction task. The radiomic-based nomogram had an internally estimated AUC of 0.9334 (95% CI 0.881‐0.968) in the capsular invasion prediction task and 0.8001 (95% CI 0.663‐0.898) in the cross-label association analysis task. In RF models, clinical indicator-based and radiomic-based RFs exhibited an AUC of 0.7646 (95% CI 0.651‐0.857) and 0.8102 (95% CI 0.703‐0.892) in the cross-label association analysis task, respectively. The NN model performed promisingly, with an AUC of 0.775 (95% CI 0.621‐0.903) in the cross-label association analysis task and a mean absolute error of <0.05 on the calibration curve.

**Conclusions:**

Capsular invasion is a strong predictor of NI risk in thyroid carcinoma. Radiomic models based solely on preoperative CT images show potential for preoperative NI risk stratification. Models incorporating clinical parameters (obtained from postoperative tissue), including the integrated multimodal model, are more accurately characterized as postoperative risk stratification tools. The NN model, which integrated raw CT images with clinical data, achieved an AUC of 0.775 (95% CI 0.621‐0.903), underscoring the potential of such multimodal analysis to capture complex relationships between imaging phenotypes and tissue-level biomarkers for enhanced postoperative assessment. This framework’s radiomic component points toward purely image-based, preoperative evaluation tools’ development.

## Introduction

Thyroid carcinoma represents the most prevalent malignancy of the endocrine system, with a worldwide increasing incidence in recent years [1]. The biological behavior of thyroid carcinoma varies considerably. Some patients experience favorable outcomes, while others face higher risks of recurrence and metastasis [2]. Accurate assessment of tumor aggressiveness and prognostic factors is critical for developing personalized therapeutic strategies and improving patients’ quality of life. Capsular invasion and neural invasion (NI) are key prognostic determinants in thyroid carcinoma. Research has demonstrated that thyroid carcinoma patients with capsular invasion or NI exhibit significantly higher postoperative recurrence and mortality rates compared to those without such invasive features [3]. According to previous studies, thyroid carcinoma biomarkers galectin-3 (Gal-3), Hector Battifora Mesothelial Epitope-1 (HBME-1), and cytokeratin 19 (CK19) are often overexpressed in malignant tumors compared to benign lesions. However, whether they are related to capsular invasion and NI is unclear [4]. Radiomics, an emerging computational technology, enables high-throughput extraction of quantitative features from medical imaging data, which reflect multidimensional tumor characteristics, including morphology (eg, ranges of tumor boundaries), texture, and functional heterogeneity [5,6]. Computed tomography (CT)–based radiomic analysis holds promise for identifying imaging biomarkers closely associated with tumor biology and may provide novel insights for diagnosis and prognosis [7,8]. The development of CT-derived radiomic biomarkers linked to capsular invasion in thyroid carcinoma may facilitate early and precise prediction of tumor aggressiveness. Furthermore, clarifying the stratification value of these biomarkers for NI risk could empower clinicians to more accurately assess risk preoperatively and provide a reliable basis for targeted neuroprotective strategies [9]. Nevertheless, current research on molecular imaging biomarkers for capsular invasion in thyroid carcinoma remains in its exploratory phase, and their potential for stratifying NI risks needs further investigation. This study aims to systematically identify CT-based radiomic biomarkers associated with capsular invasion in thyroid carcinoma and comprehensively evaluate their risk stratification utility for NI, thereby advancing novel methodologies for precision oncology in thyroid carcinoma management.

## Methods

### Study Population

A total of 111 patients diagnosed with thyroid carcinoma and hospitalized were retrospectively enrolled. Based on the presence of capsular invasion, patients were categorized into a control group (capsular invasion–negative, n=48) and a study group (capsular invasion–positive, n=63). No significant differences in baseline characteristics were observed between the 2 groups (Table 1), ensuring comparability for subsequent analyses.

**Table 1.** Comparative analysis of baseline characteristics between patients with and without membrane invasion.^a^

		Control group (n=48)	Study group (n=63)	Statistics	*P* value
Gal-3^b^, n (%)				9.3 (1)^c^	.002
	High expression	18 (37.5)	42 (66.67)		
	Low or no expression	30 (62.5)	21 (33.33)		
HBME-1^d^, n (%)				1.7 (1)^c^	.19
	High expression	45 (93.75)	62 (98.41)		
	Low or no expression	3 (6.25)	1 (1.59)		
CK19^e^, n (%)				29.3 (1)^c^	.001
	High expression	26 (54.17)	61 (96.83)		
	Low or no expression	22 (45.83)	2 (3.17)		
NI^f^, n (%)				27.9 (1)^c^	<.001
	Yes	3 (6.25)	34 (53.97)		
	No	45 (93.75)	29 (46.03)		
CEA^g^ (ng/mL), mean (SD)		31.66 (5.12)	34.91 (4.07)	−3.73（109）^h^	<.001
CCSA-2^i^ (ng/mL), mean (SD)		19.26 (4.98)	19.86 (4.65)	−0.66（109）^h^	.51
CA199^j^ (U/mL), mean (SD)		59.95 (11.03)	74.17 (10.07)	−7.07（109）^h^	<.001
CA125^k^ (U/mL), mean (SD)		77.96 (13.75)	87.12 (14.87)	−3.32（109）^h^	.001
Age (y), mean (SD)		45.29 (12.37)	44.97 (10.83)	0.99（109）^h^	.64
BMI (kg/m^2^), mean (SD)		21.83 (2.16)	22.51 (2.17)	−1.49（109）^h^	.14

a High expression was defined as ≥2-fold expression relative to peripheral blood mononuclear cell controls.

b Gal-3: galectin-3.

c Chi-square test.

d HBME-1: Hector Battifora Mesothelial Epitope-1.

e CK19: cytokeratin 19.

f NI: neural invasion.

g CEA: carcinoembryonic antigen.

h *t* test.

i CCSA-2: circulating cancer-specific antigen.

j CA199: carbohydrate antigen 199.

k CA125: carbohydrate antigen 125.

Inclusion criteria were as follows: (1) diagnosis of thyroid carcinoma confirmed by postoperative histopathological examination and compliance with the SEOM-GETNE-TTCC（ Spanish Society of Medical Oncology, Spanish Group for Neuroendocrine and Endocrine Tumors, and Spanish Group for Head and Neck Tumors） Clinical guideline for thyroid cancer (2023) [10]; (2) preoperative neck CT scan; (3) availability of complete clinical records, including postoperative histopathological reports explicitly documenting capsular invasion and NI status; and (4) high-quality imaging data with standardized acquisition protocols, clearly delineated tumor boundaries, and absence of motion artifacts. Exclusion criteria included having (1) concurrent diagnosis of other malignancies or (2) severe hepatic or renal dysfunction, active autoimmune diseases, or pregnancy or lactation.

### Ethical Considerations

This study was approved by the Ethics Committee of Air Force Medical Center, Air Force Medical University (No. 2025‐47-YJ01) and adhered to the principles outlined in the Declaration of Helsinki. The ethics committee specifically evaluated and approved a waiver of informed consent for this retrospective study, considering the use of anonymized imaging and clinical data and the impracticability of obtaining individual consent. All data were anonymized and deidentified before analysis to ensure patient privacy and confidentiality. Participants did not receive any compensation.

### Imaging Feature Extraction

CT scans were performed on 2 scanner models: a GE Discovery energy-spectrum CT (GE Healthcare) and a Siemens SOMATOM Definition Flash dual source CT ( IncSiemens Healthineers). Scanner parameters were as follows: GE parameters: 120 kV, automatic tube current (250‐465 mA), 512 × 512 matrix, pitch 0.9. Siemens parameters: 120 kV, reference 210 mAs (CARE Dose4D), 512 × 512 matrix, pitch 0.8. All scans used iohexol contrast (2 mL/kg, 3‐4 mL/s) with arterial/venous phases at 30/50 seconds. Contiguous 5 mm slices were acquired, and 1.25 mm thin-section reconstructions were generated for analysis.

For data harmonization, to mitigate interscanner variability, all images were reconstructed to a 1.25 mm slice thickness. For radiomic features (A1-A9), *z* score normalization was applied across the entire dataset. For deep learning input, minimum-maximum scaling to [0,1] was applied per scan.

Before feature extraction, raw CT images underwent preprocessing to enhance image quality and ensure accurate subsequent analyses. Thyroid cancer lesion boundaries were independently delineated by 2 associate chief physicians with 6 and 8 years of experience in head and neck imaging diagnosis, using ITK-SNAP (developed by the ITK-SNAP Development Team, University of Pennsylvania) software to manually delineate regions of interest (ROIs) on arterial phase CT images. For venous-phase features (A3, A5, A7, and A9), arterial-phase ROIs were propagated to venous-phase images using rigid image registration, followed by manual verification and adjustment. To verify consistency, CT images of 30 (27% of the cohort) randomly selected patients were analyzed. Intraclass correlation coefficients (ICC) were calculated to assess both ROI volume consistency and numerical consistency of core gray level cooccurrence matrix (GLCM) features (contrast, correlation, and energy; distance *d*=1, angle *θ*=0°, 45°, 90°, 135°). The ICC for ROI volume was 0.92 (95% CI 0.85‐0.96, *P*<.001), and the ICC value range of GLCM core features was 0.88‐0.94 (both *P*<.001), indicating excellent reproducibility. Discrepancies in 3 (10%) cases were resolved through consensus review with postoperative pathology as reference.

Dimensionality reduction was performed via principal component analysis (PCA) and linear discriminant analysis (LDA). Intensity-based features were concurrently extracted and subjected to least absolute shrinkage and selection operator (LASSO) regression to identify robust predictors. To mitigate interscan variability in gray-level intensity values caused by heterogeneous acquisition protocols, for the extraction of hand-crafted radiomic features (A1-A9), *z* score normalization was applied across the entire dataset to standardize the gray-scale distribution and eliminate interscan variability caused by heterogeneous CT acquisition protocols. It should be noted that applying normalization to the full dataset before cross-validation could lead to potential information leakage, as the mean and SD used for normalization may inadvertently include information from samples designated for validation. Although our primary aim was to compare the relative performance of different models rather than to provide absolute performance estimates, we acknowledge this methodological limitation and have further addressed its potential impact in the Discussion section. For the deep learning model (DenseNet121) input only: the original-resolution ROIs were first normalized to a [0, 1] range using minimum-maximum scaling, then resized to 224×224 pixels (to meet the fixed-size input requirement of the pretrained DenseNet121 backbone) and converted to 3-channel format by duplicating the grayscale image across RGB (red, green, and blue) channels. This 224×224 resizing step was exclusive to deep learning model training and did not involve radiomic feature extraction.

Notably on ROI resolution for feature extraction, a critical distinction was made between radiomic feature extraction and deep learning model input preparation in this study. All radiomic texture features (111 GLCM features and 9 LASSO-selected A1-A9 features) were extracted from original-resolution CT ROIs to avoid systematic scaling bias and retain the physical meaning of texture metrics. The resizing of ROIs to 224×224 pixels was a dedicated preprocessing step for the DenseNet121-based deep learning model, as pretrained convolutional neural networks (NN) typically require fixed-size inputs. This separation of technical processes ensures the reliability of both radiomic feature analysis and deep learning model training.

### Model Development

Given the limited total sample size (111 patients), to maximize the use of data, a full-sample modeling approach was adopted.

Model construction: all 111 cases were used as the analysis cohort, with capsular invasion status (1=capsular invasion–positive, n=63; 0=capsular invasion–negative, n=48) as the labeling variable. Input features were tailored to each model type: a common set of clinical biomarkers (for the complete list, see Table 1) was used across all models, while pixel-level CT features A1-A9 (selected via LASSO regression from GLCM texture features of CT ROIs) were used specifically in the traditional radiomic models (nomogram and random forest [RF]).

The NN model used a multimodal input, comprising both raw arterial-phase CT ROI images and the suite of clinical biomarkers (for the complete list, see Table 1), to autonomously learn high-level spatial and clinical patterns associated with capsular invasion (Multimedia Appendix 1). It is crucial to note that the hand-crafted radiomic features (A1-A9) were not direct inputs to this NN model. For each patient, a single 2D axial slice with the largest tumor cross-section was selected, standardized to 224×224 pixels, and converted to 3-channel format. Rationale for arterial-phase only input: this single-phase design was implemented for two primary reasons: (1) to maintain model simplicity and mitigate overfitting risk given the limited sample size, and (2) because the arterial phase provides the most direct imaging correlate of tumor vascularity, a key biological process underlying invasion. We hypothesized that the convolutional NN could learn high-level features from the arterial phase that are informative for predicting outcomes potentially associated with venous-phase patterns. Rationale despite venous-phase radiomic feature importance: it is noteworthy that in our traditional radiomics analysis, a feature derived from the venous phase (A9) showed high importance. The decision to use only arterial-phase images for the deep learning model was based on a complementary hypothesis: that the convolutional NN could learn high-level, abstract features from the arterial phase that are biologically or spatially correlated with the tumor properties captured by the venous-phase radiomic signature (A9). This approach allowed us to test whether a single, routinely acquired phase could provide sufficient information for the NN to make accurate predictions, thereby simplifying the model and its potential clinical translation.

Concerning model validation, NI status (1=NI-positive, n=37; 0=NI-negative, n=74) was used for a cross-label association analysis. The core goal was to evaluate whether the features associated with capsular invasion (as captured by our models) were also rank-informative for NI status within the same cohort. The full 111-case dataset was used for both (1) model training (with capsular invasion status as the label) and (2) cross-label association evaluation (with NI status as the label). It is crucial to note that this analysis evaluates the association between capsular invasion–related features and NI status on the same samples, primarily reflecting their strong pathological correlation rather than constituting an independent predictive validation on unseen data. The full 111-case cohort, labeled based on the presence or absence of capsular invasion, was used to construct 3 predictive models (nomogram, RF, and NN) to capture capsular invasion–associated features. The same full cohort, labeled based on the presence or absence of NI, was then used to evaluate the models’ cross-predictive performance for NI risk stratification. To address potential concerns regarding the unconventional data usage design and to empirically validate our model’s generalizability, we performed a supplementary traditional 7:3 hold-out validation.

For the nomogram development, clinically significant variables associated with capsular invasion were incorporated into a clinical nomogram, while gray-level features selected via LASSO regression were integrated into an imaging-based nomogram. Calibration curves (predicted probability vs observed probability) were generated to assess agreement between model predictions and observed outcomes, with internal validation performed using a bootstrap resampling method (B=1000 iterations).

The RF model architecture comprised 2 components: a clinical RF model incorporating capsular invasion–associated variables and an imaging RF model integrating LASSO-selected radiomic features. Hyperparameter optimization was conducted via 5-fold cross-validation using the caret package, with the number of trees (ntree) tuned from 50 to 500 (step size=5) and the minimum number of features per node split (mtry) ranging from 2 to 10. Model interpretability was enhanced through feature importance plots, decision tree visualization, receiver operating characteristic (ROC) curves, and out-of-bag error analysis.

For the input of the NN model (based on DenseNet121): to comply with the fixed-size input requirement of the pretrained DenseNet121 backbone, the original-resolution ROIs were first normalized to a [0, 1] range using minimum-maximum scaling, then resized to 224×224 pixels. The single-channel grayscale images were further converted to a 3-channel format by duplication across RGB channels. The first 10 convolution blocks of DenseNet121 were reserved for extracting high-level image features, and the feature map was compressed into fixed-length vectors through global average pooling. Image feature vectors (extracted by DenseNet121) and clinical feature vectors were normalized, respectively, and directly spliced into multimodal feature vectors. The spliced features were input to the fully connected layer and were finally classified (capsular invasion: positive or negative) through the Sigmoid output layer. The 9 radiomic features (A1-A9) were selected from GLCM features of the ROI via LASSO regression. The model was optimized using the Adam optimizer (learning rate=1×10⁻⁴) with binary cross-entropy loss over 50 training epochs (batch size=16). Early stopping was triggered if validation loss plateaued for 10 consecutive epochs (MultimediaAppendices 1  2).

**Figure 1.**
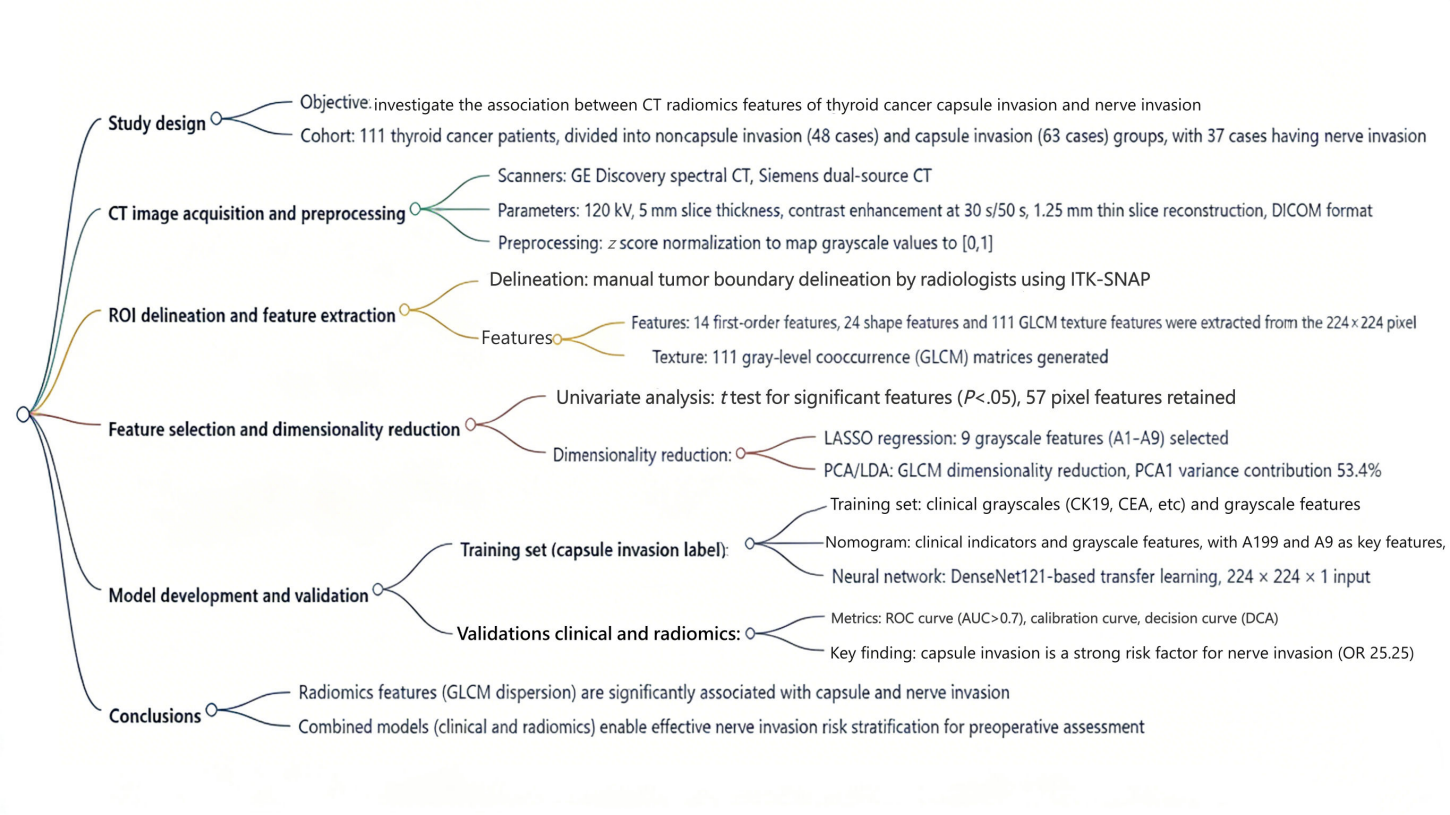
Workflow of CT imaging feature extraction and predictive model development. A9: venous-phase coefficient of variation; AUC: area under the curve; CA199: carbohydrate antigen 199; CEA: carcinoembryonic antigen; CK19: cytokeratin 19; CT: computed tomography; DCA: decision curve analysis; DICOM: Digital Imaging and Communications in Medicine; GE: General Electric; GLCM: gray-level co-occurrence matrix; LASSO: least absolute shrinkage and selection operator; LDA: linear discriminant analysis; OR: odds ratio; PCA: principal component analysis; ROC: receiver operating characteristic; ROI: regions of interest.

The technical workflow is illustrated in Figure 1.

### Model Interpretability Analysis

**Figure 2.**
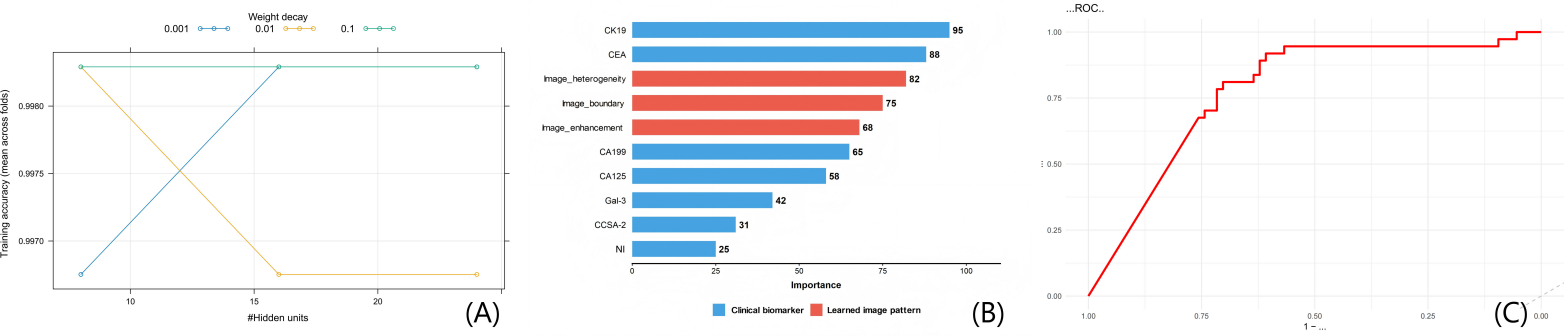
Neural network model performance. (A) Mean training accuracy curves across the 5-fold cross-validation splits, plotted for different numbers of hidden units. (B) SHAP-based feature importance (normalized 0-100). Blue: clinical biomarkers (direct inputs); red: image patterns (learned from raw CT). (C) ROC curve for neural invasion prediction. Note for (B): normalized importance scores derived from SHAP analysis (see subsection in Methods: Model interpretability analysis). Imaging patterns were semantically annotated based on activation map visualizations. CA125: carbohydrate antigen 125; CA199: carbohydrate antigen 199; CCSA-2: circulating cancer-specific antigen; CEA: carcinoembryonic antigen; CK19: cytokeratin 19; CT: computed tomography; Gal-3: galectin-3; NI: neural invasion; NN: neural network; ROC: receiver operating characteristic; SHAP: Shapley Additive Explanations.

To interpret the NN’s predictions and derive the importance scores in Figure 2B, we used Shapley Additive Explanations (SHAP). The analysis pipeline was as in the following paragraphs.

Concerning calculation for the validation set, SHAP values were computed for each input feature (clinical variables and the high-dimensional features extracted from raw CT images by DenseNet121). These values quantify a feature’s contribution to a specific prediction.

For aggregation and semantic mapping, global importance for each clinical variable was defined as its mean absolute SHAP value. For image features, activation maps corresponding to features with high mean absolute SHAP values were analyzed. Recurring visual patterns were inductively categorized into 3 semantic concepts: “Image_Heterogeneity,” “Image_Boundary,” and “Enhancement_Dynamics.” The importance score for each concept was calculated as the mean absolute SHAP value of its constituent image feature dimensions.

In normalization, all resulting scores (for clinical variables and image concepts) were linearly normalized to a 0‐100 scale for comparative visualization in Figure 2B.

### Data Collection

All thyroid carcinoma tissue specimens were collected immediately after surgical resection, and 10% neutral buffered formalin was used for fixation within 30 minutes for morphological preservation. After paraffin embedding, 5-μm-thick sections were prepared, and tumor tissue areas (with tumor cell content ≥80%, confirmed by 2 pathologists) were microdissected for quantitative polymerase chain reaction (qPCR) analysis. This ensured that the detected expression of Gal-3, HBME-1, and CK19 accurately reflected the biological characteristics of thyroid carcinoma cells.

Real-time fluorescence qPCR was performed to measure the levels of Gal-3, HBME-1, and CK19 in thyroid carcinoma tissue specimens from the 111 enrolled patients. Glyceraldehyde-3-phosphate dehydrogenase served as the endogenous reference gene for normalization. Peripheral blood mononuclear cells (PBMCs) from age- and sex-matched healthy individuals undergoing routine health screenings were used as the control group for qPCR analysis. Relative gene expression levels ≥2-fold compared to PBMC controls were pragmatically defined as high expression for subsequent categorical analysis. It is important to note that the use of PBMCs as a control group is a methodological consideration due to the practical and ethical challenges in obtaining matched healthy thyroid tissue samples. As PBMCs (of nonepithelial origin) and thyroid epithelial cells have fundamentally different baseline gene expression profiles, this comparison is intended to establish a highly sensitive detection threshold for identifying genes that are actively expressed at levels substantially above this technical background in tumor tissue, rather than to provide precise biological fold-change values across tissue types. The ≥2-fold threshold therefore serves as a pragmatic cutoff for distinguishing tumors with detectably high expression of the target genes. Therefore, it is crucial to emphasize that “high expression” as defined in this study indicates detectability above a nonepithelial (PBMC) baseline, and should not be interpreted as pathological overexpression relative to normal thyroid tissue.

Concurrently, baseline characteristics and serum levels of tumor biomarkers, including carcinoembryonic antigen (CEA), carbohydrate antigen 199 (CA199), and carbohydrate antigen 125 (CA125), and the second-generation assay for circulating cancer-specific antigen, were systematically collected.

### Statistical Methods

All statistical analyses were performed using SPSS Statistics (version 27.0; IBM Corp) and R software (version 4.4.3; R Foundation). Continuous variables with normal distribution were expressed as mean ± SD ($\overset{-}{\text{x}}$± s), while categorical data were presented as n (%). Intergroup comparisons were conducted using independent samples *t* tests for continuous variables and chi-square tests for categorical variables. Multivariable binary logistic regression analysis was used to identify independent risk factors. The RF model was developed using the randomForestSRC, *ggRandomForests*, *pdp*, and *GGally* packages in R. Nomogram construction and calibration were implemented with the *rms*, *dcurves*, and *pROC* packages in R. NN architecture was built using the keras interface in R. Model accuracy was evaluated via the confusionMatrix() function from the *caret* package. All visualizations were generated using the *ggplot2* package in R. A *P* value <.05 was considered statistically significant.

## Results

### Overview

In this section, Table 1 presents the baseline characteristics of patients. The detailed multivariate logistic regression results (originally Tables 2 and 3) have been moved to the supplementary material; see Tables S1 and S2 in Multimedia Appendix 3.

### Baseline Characteristics Comparison

**Figure 3.**
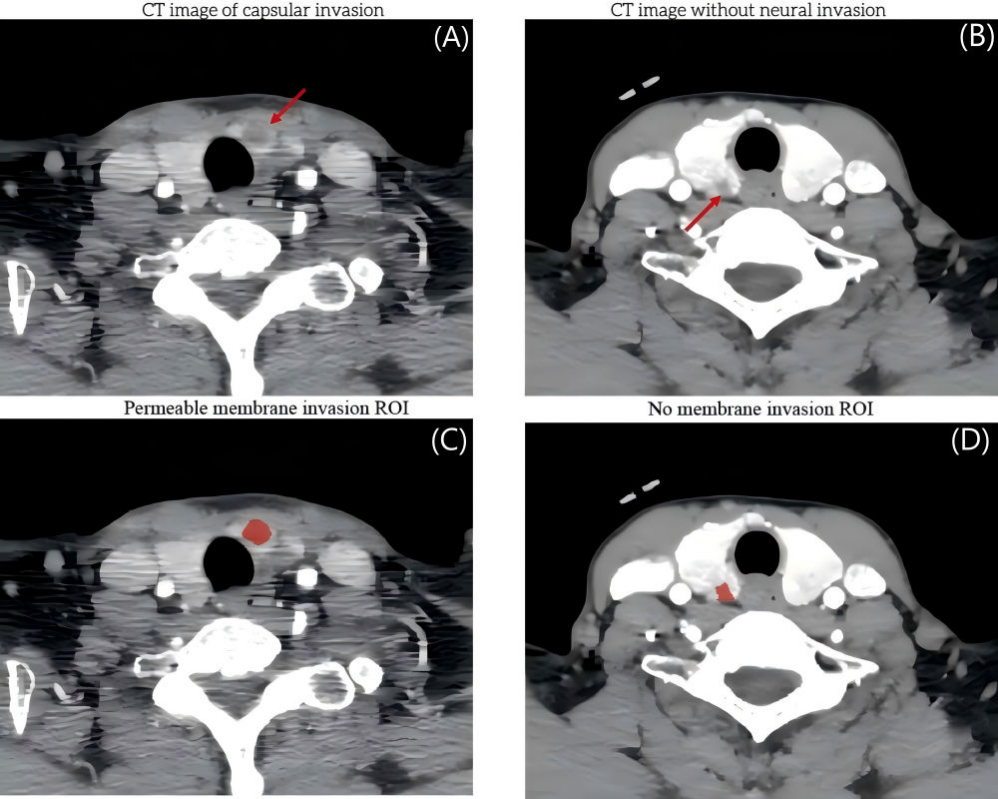
Representative arterial-phase CT images of thyroid carcinoma. (A-C) A case with pathologically confirmed capsular invasion showing irregular tumor margins (arrow). (B-D) A case without NI demonstrating well-defined tumor boundaries. CT: computed tomography; NI: neural invasion; ROI: regions of interest.

The comparative analysis of baseline characteristics between patients with and without capsular invasion revealed statistically significant differences in the categorized expression levels (high vs low or no expression) of Gal-3 and CK19, as well as in the serum concentrations of tumor markers including CEA, CA199, and CA125 (*P*<.05, Table 1). Representative arterial-phase CT images and corresponding ROI delineations from patients with thyroid carcinoma are shown in Figure 3A-D.

### GLCM Analysis

**Figure 4.**
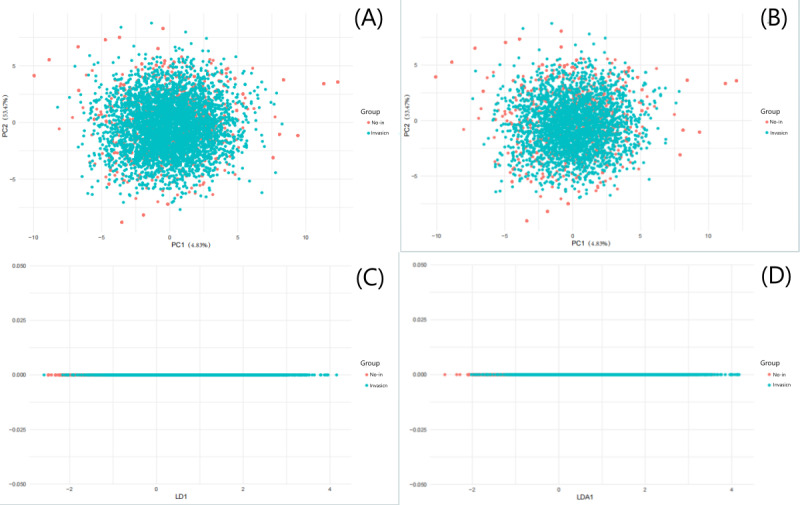
Dimensionality reduction analysis. (A) PCA of capsular invasion status; (B) PCA of NI status; (C) LDA of capsular invasion status; (D) LDA of NI status. LD1: linear discriminant 1; LDA: linear discriminant analysis; LDA1: linear discriminant 1; NI: neural invasion; no-in: noninvasion; PC1: principal component 1; PC2: principal component 2; PCA: principal component analysis.

The GLCM analysis yielded 111 texture features, which were subsequently subjected to dimensionality reduction via PCA and LDA. A total of 14 principal components were extracted for capsular invasion classification, with PCA1 and PCA2 accounting for 53.47% and 4.83% of the total variance, respectively. Significant intergroup differences were observed in PCA1 (*t*_109_=11.671, *P*=.001, 2-tailed), where patients without capsular invasion exhibited greater dispersion in GLCM patterns, as evidenced by their larger Euclidean distance (6.98 vs 4.12 in the capsular invasion group). Similarly, NI analysis demonstrated significant differences in both PCA1 (*t*_109_=10.339, *P*=.001) and PCA2 (*t*_109_=−2.0187, *P*=.04). Non-NI cases again displayed greater GLCM dispersion, with Euclidean distances measuring 7.02 vs 4.05 in the NI group, a pattern consistent with the capsular invasion findings. LDA projections further corroborated this dispersion pattern, showing broader distribution clusters in noninvasion groups compared to their invasion counterparts. Visual representations of these analytical outcomes are provided in Figure 4.

### Multivariate Binary Logistic Regression Analysis Based on Clinical Indicators

It is important to emphasize that the following multivariate logistic regression analyses are intended to explore factors associated with the co-occurrence of pathological features, rather than to build preoperative predictive models. As both capsular invasion and NI are postoperative pathological diagnoses, their inclusion as variables in models predicting each other primarily reflects their strong pathological correlation, not a causal predictive relationship usable for preoperative assessment.

A multivariate binary logistic regression model was constructed with capsular invasion status as the dependent variable (1=presence, 0=absence). The analysis identified CK19, CEA, CA199, CA125, and NI as significant independent risk factors for capsular invasion. Notably, CK19 demonstrated an exceptionally strong association with capsular invasion, yielding an odds ratio (OR) of 60.491 (*P*<.05, see Table S1 in Multimedia Appendix 3).

A parallel analysis was performed with NI status as the dependent variable (1=presence, 0=absence). Among all clinical indicators, only capsular invasion emerged as a significant predictor of NI, with an OR of 25.25 (*P*<.05, Table S2 in Multimedia Appendix 3), underscoring the pronounced influence of capsular invasion on NI risk.

### Gray-Level Feature Selection via LASSO Regression Analysis

**Figure 5.**
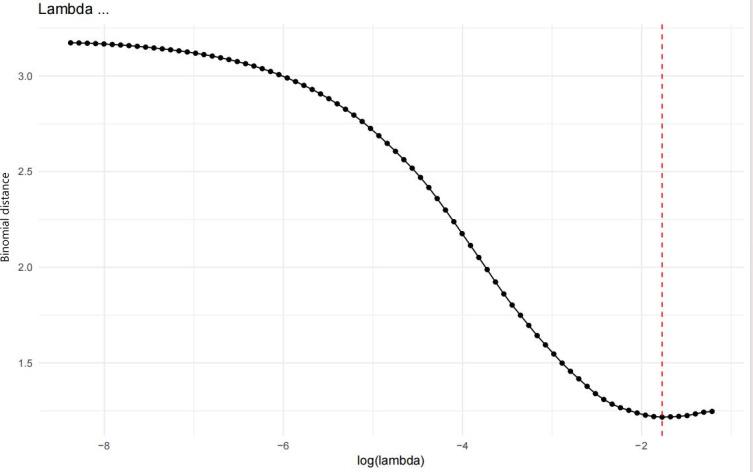
Cross-validated deviance profile versus log(λ) values.

A set of radiomic features was extracted from the pixels within the ROI. Following exclusion of features without significant differential expression, 57 discriminative features were retained for LASSO regression analysis. During variable selection, the penalty coefficient (λ) was iteratively adjusted to compress the initial 57 predictors. Optimal λ value (λ=0.017) was determined through cross-validation, achieving minimal model deviance while balancing parsimony and predictive accuracy. This process yielded a refined model incorporating 9 pivotal features (designated A1-A9), as presented in Figure 5. The 9 pixel-level features (A1-A9) selected in this study directly reflect the heterogeneity of contrast enhancement and tissue composition of thyroid carcinoma on CT images. Specifically: (1) differences in arterial-phase pixel intensity mean (A1) reflect variations in tumor vascular density: tumors with higher A1 values (stronger arterial enhancement) are more likely to have capsular invasion/NI, as increased angiogenesis provides conditions for tumor invasion; (2) variations in pixel intensity SD (A2) correspond to intratumoral necrosis: higher A2 values (uneven enhancement) often indicate necrotic foci, which are associated with poor differentiation (CK19 positivity) and higher NI risk; and (3) changes in venous-phase pixel percentile (A3 and A8) reflect capsule integrity: lower A3 or A8 values suggest capsule rupture, a direct marker of capsular invasion. These imaging phenotypes serve as quantifiable surrogates for pathological characteristics, such as vascular density, necrosis, and capsular status, enabling noninvasive preoperative assessment of tumor aggressiveness. Detailed feature definitions are provided in Multimedia Appendix 2, with additional descriptions in Multimedia Appendix 1.

### Development of Nomogram Models for Predicting NI Based on Capsular Invasion

**Figure 6.**
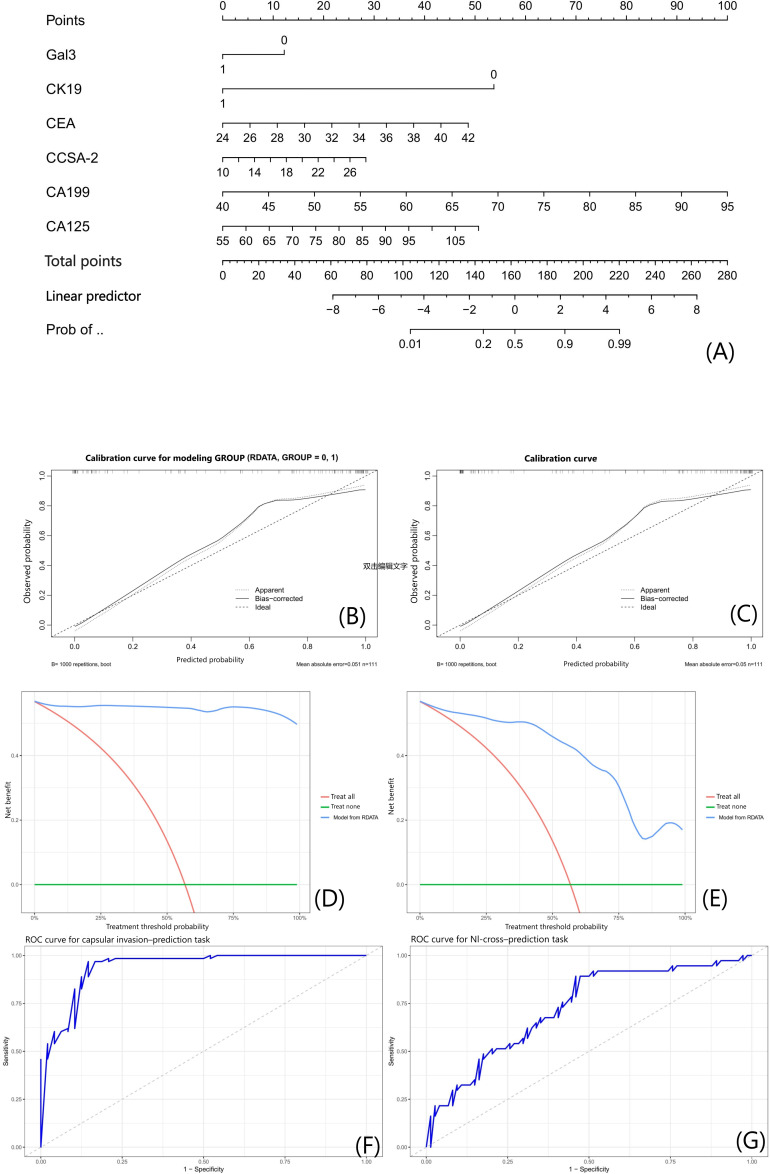
Clinically parameterized nomogram model for neural invasion prediction. (A) Nomogram integrating clinical biomarkers (Gal-3, CK19, CEA, CA199, and CA125) to predict NI risk, with key variables (CK19 and CA199) marked by “*” (high OR values from Tables S1 and S2 in Multimedia Appendix 3); red dashed lines guide point assignment for each variable. (B and C) Calibration curves for the capsular invasion–prediction task ((B) modeled on full 111-case dataset) and the cross-label association analysis task ((C) cross-prediction on full 111-case dataset), showing agreement between predicted NI probability (x-axis) and observed NI status (y-axis); 95% CI bands (light blue or red) and MAE are labeled. (D and E) DCA curves for both tasks (using the full 111-case dataset, with no traditional train or test split), comparing the net benefit of the nomogram (dark blue line) with “treat all” (red line) and “treat none” (gray line) strategies across different NI risk thresholds. (F and G) ROC curves for the capsular invasion-prediction task ((F) modeled on full 111-case dataset) and the cross-label association analysis task ((G) evaluated on full 111-case dataset), with exact AUC values and 95% CIs labeled; light blue or red bands represent 95% CIs. All analyses were based on the full 111-case cohort (capsular invasion–labeled modeling and NI-labeled evaluation). AUC: area under the curve; CA125: carbohydrate antigen 125; CA199: carbohydrate antigen 199; CCSA-2: circulating cancer-specific antigen; CEA: carcinoembryonic antigen; CK19: cytokeratin 19; DCA: decision curve analysis; Gal-3: galectin-3; MAE: mean absolute error; NI: neural invasion; OR: odds ratio; Prob: probability; RDATA: research data; ROC: receiver operating characteristic; .

A tumor biomarker-derived nomogram was constructed to assess the predictive value of capsular invasion for NI. ROC curve analysis demonstrated robust discriminative performance, with an internally estimated area under the curve (AUC) of 0.9418 for the capsular invasion-prediction task (modeled on the full 111-case dataset) and 0.7253 for the cross-label association analysis task (evaluated on the full 111-case dataset). Calibration curves indicated high consistency between predicted probabilities and observed outcomes across both tasks. Decision curve analysis further validated the clinical utility of the model, as its net benefit across all threshold probabilities outperformed the “treat-all” and “treat-none” strategies (Figure 6).

**Figure 7.**
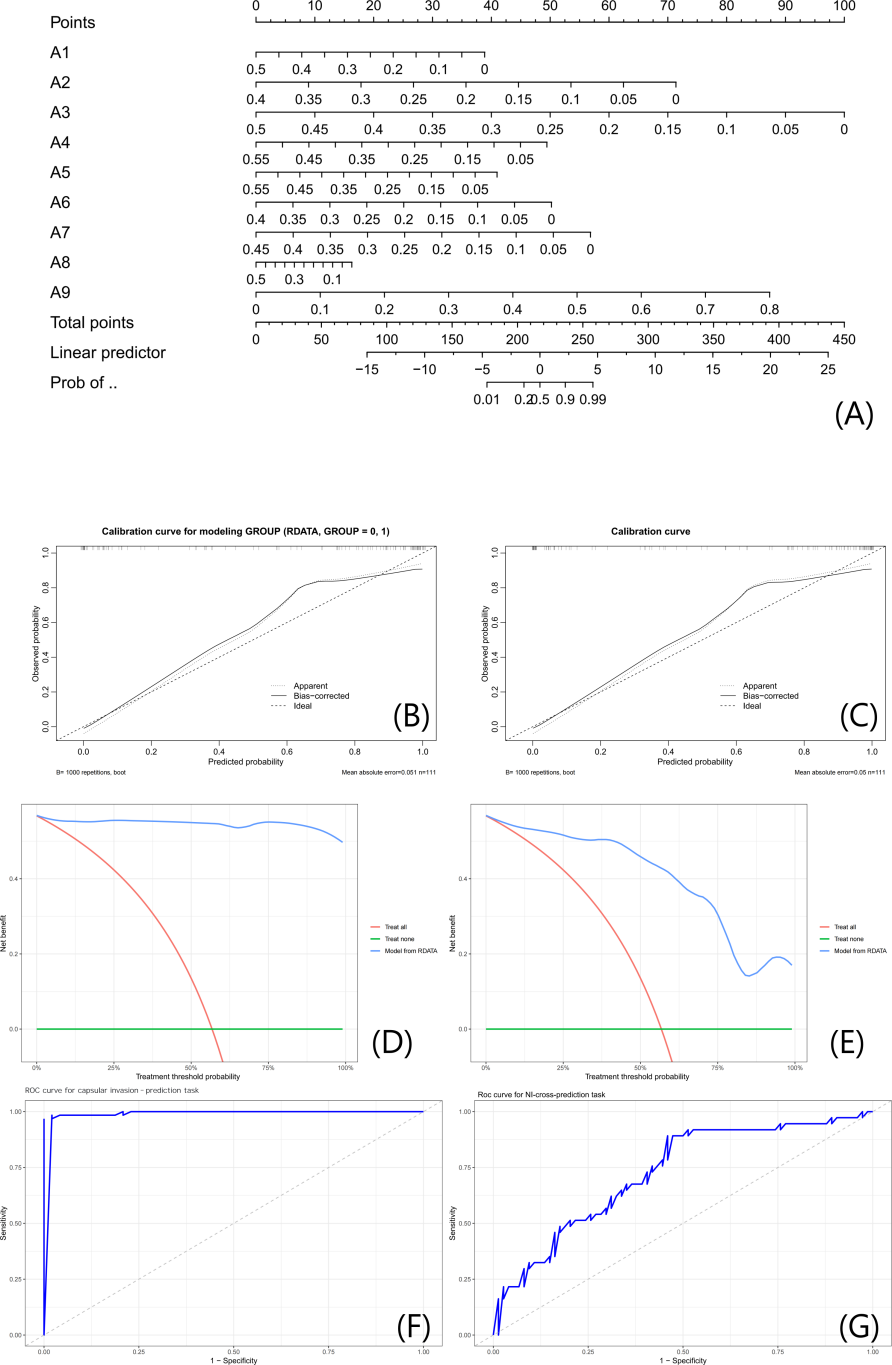
Gray-level feature-integrated nomogram model for neural invasion prediction. (A) Nomogram integrating LASSO-selected pixel-level features (A1-A9) to predict NI risk; footnotes define key features (A1 and A9) per the Gray-Level Feature Selection via LASSO Regression Analysis section. (B and C) Calibration curves for capsular invasion-prediction task ((B) modeled on full 111-case dataset) and cross-label association analysis task ((C) evaluated on full 111-case dataset); hollow circles (dark blue) and solid squares (coral red) represent data points, with 95% CI bands and MAE ((B) 0.05; (C) 0.049) labeled. (D and E) DCA curves, with the nomogram (dark blue line) showing higher net benefit than reference strategies across NI risk thresholds. (F and G) ROC curves for the capsular invasion-prediction task ((F) modeled on full 111-case dataset) and the cross-label association analysis task ((G) evaluated on full 111-case dataset); exact internally estimated AUC values (capsular invasion-prediction task: 0.9334; cross-label association analysis task: 0.8001) and 95% CIs are labeled, with light blue or red bands representing 95% CIs. All analyses used the full 111-case cohort (capsular invasion–labeled modeling and NI-labeled evaluation). AUC: area under the curve; DCA: decision curve analysis; LASSO: least absolute shrinkage and selection operator; MAE: mean absolute error; NI: neural invasion; Prob: probability; RDATA: research data.

A parallel nomogram model incorporating gray-level radiomic features achieved internally estimated AUC values of 0.9334 for the capsular invasion-prediction task (full 111-case dataset) and 0.8001 for the cross-label association analysis task (full 111-case dataset). Calibration curves similarly confirmed strong predictive accuracy for both tasks, and decision curve analysis revealed that the model achieved superior net clinical benefit across the entire spectrum of risk thresholds compared to reference strategies (Figure 7).

### RF Model Development

**Figure 8.**
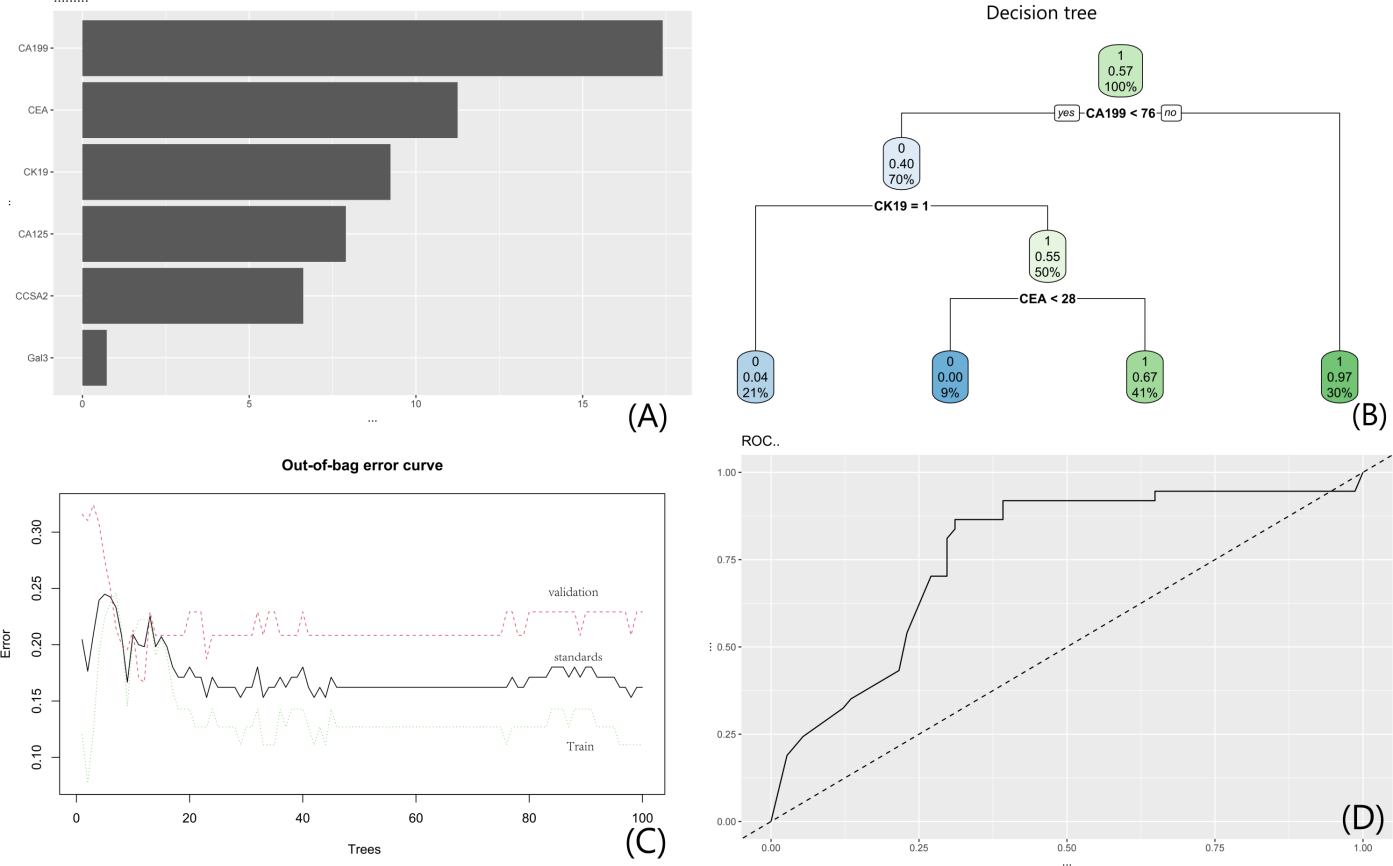
RF model based on clinical biomarkers. (A) Feature importance plot; (B) a representative surrogate tree from the RF ensemble; (C) out-of-bag error trajectories; and (D) ROC curve. CA125: carbohydrate antigen 125; CA199: carbohydrate antigen 199; CCSA2: colon cancer-specific antigen-2; CEA: carcinoembryonic antigen; CK19: cytokeratin 19; Gal3: galectin-3; RF: random forest; ROC: receiver operating characteristic.

An RF model was constructed using tumor biomarkers, with CA199 demonstrating the highest feature importance (visualized in Figure 8A). A representative surrogate tree from the RF ensemble (visualized in Figure 8B) initiated classification through sequential partitioning: the root node split on “CA199<76,” followed by a “yes” branch subdivided by “CK19=1,” and further partitioned via “CEA<28.” Out-of-bag error curves (visualized in Figure 8C) revealed stabilized misclassification rates for both training and validation sets as tree numbers increased, with training set errors consistently exceeding validation set values. The 5-fold cross-validation AUC (for capsular invasion prediction) was 0.782 (95% CI 0.671‐0.874), and the out-of-bag error rate stabilized at 0.213 when ntree ≥300 (visualized in Figure 8D). When this model was used to predict NI (full-sample NI label), the model exhibited discriminative efficacy with an AUC of 0.7646, only 2.5% lower than the cross-validation AUC for capsular invasion, confirming no significant overfitting (Figure 8).

**Figure 9.**
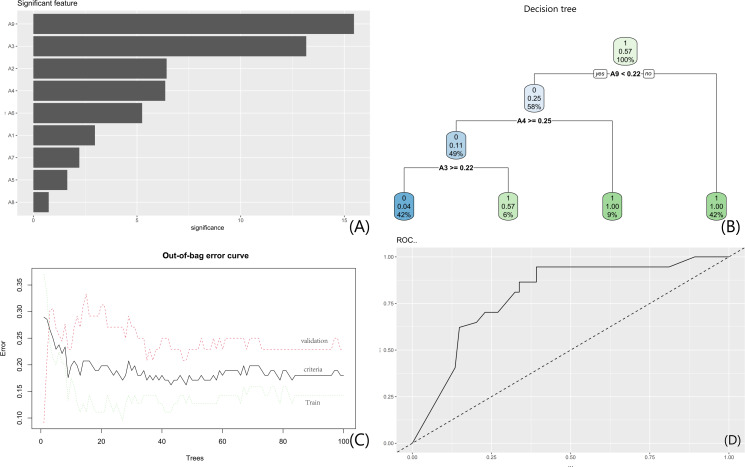
RF model based on gray-level features. (A) Feature importance plot (features A1-A9 represent the 9 key radiomic grayscale features selected via LASSO regression from GLCM texture features of CT regions of interest. They quantify tumor heterogeneity in arterial and venous phases (eg, A1, A9); (B) a representative surrogate tree from the RF ensemble; (C) out-of-bag error trajectories; (D) ROC curve. A1: arterial-phase mean intensity; A9: venous-phase coefficient of variation; CT: computed tomography

A parallel RF model incorporating gray-level features identified feature A9 as the primary contributor (corresponding to Figure 9A). A representative surrogate tree from the RF ensemble (corresponding to Figure 9B) partitioned data at “A9 <0.22,” with subsequent splits at “A4 ≥0.25” and “A3 ≥0.22.” Out-of-bag error curves mirrored the tumor biomarker-based model, showing convergence to stability with increasing tree numbers (corresponding to Figure 9C). The 5-fold cross-validation AUC (for capsular invasion prediction) was 0.825 (95% CI 0.723‐0.906), and the out-of-bag error rate stabilized at 0.187 when ntree ≥350. ROC analysis confirmed enhanced predictive performance (corresponding to Figure 9D); its AUC for NI prediction was 0.8102%‐1.8% lower than the capsular invasion cross-validation AUC, further confirming low overfitting risk (Figure 9). These data collectively validate the clinical utility of both RF models.

### NN Model Development

The NN model used exclusively two input modalities: (1) raw arterial-phase CT ROI images and (2) clinical biomarkers. No hand-crafted radiomic features (A1-A9) were directly input to the NN.

An NN model integrating multimodal data demonstrated high learning efficiency, with training accuracy values exceeding 0.99 across learning curves (Figure 2A). It is important to note that Figure 2A displays the mean training accuracy across the 5-fold cross-validation splits, not a cross-validated test accuracy. The significant gap between the near-perfect training accuracy (>0.99) and the more moderate cross-validation AUC for the primary capsular invasion prediction task (0.798) is indicative of overfitting, a common challenge in training complex models on limited datasets. While early stopping was used to mitigate overtraining by halting training when validation loss plateaued, this gap suggests that the model still learned patterns highly specific to the training samples. The architecture used hidden units ranging from 10 to 30, where variations in hidden unit counts under distinct weight decay coefficients differentially influenced cross-validated accuracy values. Feature importance analysis based on SHAP values (as detailed in the Model Interpretability Analysis section) revealed that clinical biomarkers were the most influential predictors for NI risk, particularly CK19 (normalized importance score: 95) and CEA (normalized importance score: 88; Figure 2B). Concurrently, the semantically annotated image patterns—“Image_Heterogeneity” (score: 82), “Image_Boundary” (score: 75), and “Enhancement_Dynamics” (score: 68), which were autonomously learned and inductively summarized by the NN from the raw CT inputs, also contributed substantially to the predictions. Importantly, these image patterns were learned directly from raw CT images and are conceptually distinct from the hand-crafted radiomic features (A1-A9) used in traditional radiomic models. The model achieved an AUC of 0.775 in the validation set, confirming its satisfactory generalizability beyond the training cohort. The 5-fold cross-validation AUC (for capsular invasion prediction) of the integrated NN model was 0.798 (95% CI 0.695‐0.883), with a coefficient of variation of 7.2% across folds—indicating stable performance. Early stopping was triggered at an average of 32 (SD 2.8, range: 28‐35 epochs) across the 5 folds, effectively preventing overtraining. When predicting NI (full-sample NI label), the model achieved an AUC of 0.775 (95% CI 0.621‐0.903)—only 2.3% lower than the capsular invasion cross-validation AUC, indicating that the model’s predictive ability for NI remains robust despite some degree of overfitting on the primary capsular invasion task. Detailed architectural and performance metrics are illustrated in Figure 2. Feature importance analysis within the NN identified that its predictions were driven by several key learned representations: an image pattern capturing intratumoral heterogeneity, a feature related to venous-phase enhancement dynamics, and another reflecting textural uniformity, in conjunction with the clinical biomarkers CK19 and CEA. Feature importance analysis indicated that intratumoral heterogeneity was the most influential learned representation (importance score 0.82, full score=1.0).

About validation on an internal hold-out set, to further assess generalizability, a traditional 7:3 hold-out validation was performed. The RF model based on radiomic features (A1-A9) was trained on a subset of 78 cases and was subsequently evaluated on the separate, internal hold-out set of 33 cases. The observed AUC of 0.827 was promising, but the extremely wide 95% CI (0.652‐0.981) indicates substantial uncertainty due to the small test sample size (n=33), necessitating cautious interpretation of the model’s generalizability. This additional validation yielded a promising but variable performance (AUC=0.827, 95% CI 0.652‐0.981) for NI prediction (see MultimediaAppendices 1  4 for details).

## Discussion

### Research Background and Basis

Thyroid carcinoma, the most prevalent malignancy of the head and neck region, has exhibited a rapid surge in incidence over the past decade, emerging as a critical threat to global human health [11]. Beyond its capacity to disrupt thyroid function and induce hormonal dysregulation, thyroid carcinoma frequently metastasizes to adjacent cervical tissues, lymph nodes, and even distant organs such as the lungs, liver, and bones, severely compromising physiological integrity [12]. Equally concerning are its profound psychosocial impacts, as patients endure debilitating fear, anxiety, and uncertainty regarding prognosis and treatment outcomes [13]. Current advances in thyroid carcinoma research have identified molecular markers such as *BRAF* (OMIM 164757) and *NRAS* (OMIM 164790) gene mutations, which have emerged as critical tools for diagnosis, prognostic stratification, and therapeutic decision-making [14]. Nevertheless, significant gaps persist in risk stratification for capsular invasion and NI, 2 pivotal prognostic determinants. Capsular invasion serves as a key predictor of lymph node metastasis, whereas NI directly correlates with postoperative survival quality and recurrence risk [3]. Elucidating the relationship between capsular invasion and NI is therefore essential for precise clinical assessment and optimized treatment planning. Therefore, the identification of radiogenomic biomarkers for capsular invasion using CT radiomics and the evaluation of their stratification value for NI are both scientifically imperative and clinically urgent.

### Identification of Radiomic Signatures for Invasion Risk

In this study, GLCM analysis of CT images from thyroid carcinoma patients revealed distinct radiomic signatures between tumors with and without capsular invasion or NI. A total of 111 GLCM features were extracted and subjected to dimensionality reduction via PCA and LDA, which uncovered critical associations between key imaging biomarkers and invasion risk. The heterogeneous distribution of GLCM features in noninvasive cohorts, characterized by greater dispersion, reflects heightened spatial heterogeneity in gray-level pixel relationships within tumor microenvironments. This phenomenon may correlate with histopathological characteristics such as tumor boundary demarcation and cellular alignment integrity, a concept supported by the work of Wei et al [11], who demonstrated that radiomic features from multiparametric magnetic resonance imaging could effectively predict extrathyroidal extension in papillary thyroid carcinoma by capturing similar underlying tumor heterogeneity. In contrast, tumors with capsular invasion demonstrate enhanced propensity for boundary breaching [15], resulting in homogenization of imaging features. Noninvasive lesions, by comparison, maintained relatively intact tissue architecture [16,17], which manifests as increased textural complexity on imaging. This inverse relationship is further corroborated by a recent meta-analysis [18], which confirmed that textural homogenization in medical imaging often reflects the loss of normal tissue architecture due to invasive growth patterns. Thus, these findings suggest that GLCM feature dispersion may serve as a universal imaging biomarker of tumor invasiveness [19].

Mechanistically, tumors with NI exhibit the proliferative spread of malignant cells along nerve bundles [20], resulting in textural homogeneity on imaging. In contrast, noninvasive lesions present greater histological heterogeneity in interstitial architecture and cellular distribution [21]. Although direct radiomic studies on NI in thyroid carcinoma are scarce, the work of Mu et al [22] reinforces this biological rationale by showing that a radiomic signature on CT could effectively detect metastatic lymph nodes in thyroid carcinoma, demonstrating that imaging features can capture the tumor’s propensity for local invasion and spread. LDA further corroborated these findings, demonstrating broader feature dispersion in noninvasive groups and supporting PCA findings, which suggest the utility of radiomic heterogeneity as a quantifiable biomarker for invasion risk in thyroid carcinoma [18].

### Key Molecular Markers and Risk Pathways for Invasion

Notably, 3 (6.25%) patients in the capsular invasion–negative group were pathologically diagnosed with NI. This observation indicates that while capsular invasion is a dominant driver, NI can occur through alternative pathways (eg, direct perineural infiltration). Moreover, multivariate binary logistic regression identified CK19, CEA, CA199, CA125, and NI as independent risk factors for capsular invasion. Notably, CK19, a member of the cytokeratin family, exhibited an OR of 60.491, indicating that patients with significant CK19 expression had approximately 60.5 times the odds of having capsular invasion compared to those with nonsignificant expression. This finding is consistent with prior studies reporting CK19 overexpression in papillary thyroid carcinoma [23], where its upregulation enhances tumor cell proliferation and invasive potential via disruption of intercellular adhesion molecules, thereby facilitating tumor penetration through capsular barriers [24]. Laishram et al [25] further identified CK19 as a highly sensitive and specific immunohistochemical marker for thyroid carcinoma in a cross-sectional study using histopathology as the reference standard. Elevated serum levels of CEA, CA199, and CA125 further signify increased tumor burden and epithelial-mesenchymal transition, synergistically promoting invasive phenotypes [26,27]. Intriguingly, NI itself emerged as a covariate for capsular invasion, implying shared molecular pathways or microenvironmental crosstalk. Conversely, when NI was modeled as the dependent variable, only capsular invasion remained significant, underscoring its role as a dominant predictor for NI. This finding is in line with the anatomical invasion pathway of thyroid carcinoma. Following capsular penetration, tumors demonstrate increased propensity for either direct NI (eg, recurrent laryngeal nerve involvement) or chemokine-mediated perineural infiltration through paracrine signaling modulation [28]. The resultant loss of tumor boundary integrity and disruption of the local microenvironment may jointly provide a structural basis and inflammatory background for NI. Importantly, the exclusion of variables such as age and tumor size from the model highlights the preeminence of capsular status in neural risk stratification, offering clinicians an actionable biomarker for preoperative assessment.

### Predictive Model Construction and Clinical Translation Value

For preoperative assessment, the radiomic model prioritizes spatial heterogeneity derived from preoperative CT, enabling noninvasive risk prediction via texture features [6]. Such insights may guide surgical planning, such as the extent of resection or the consideration of intraoperative nerve monitoring. For postoperative stratification, the clinical biomarker-based nomogram and the integrated multimodal model use data available after surgery. They emphasize the biological aggressiveness associated with capsular invasion, aiding in postoperative pathological substaging and recurrence risk prediction. For instance, concurrent positivity for CA199 and CK19 in patients with capsular invasion could signal high-risk subgroups for NI, indicating the need for intensified adjuvant therapy. Specifically, by integrating quantitative molecular data with imaging phenotypes, these postoperative models could provide a supplementary risk assessment that might identify patients with occult invasive features or a higher propensity for recurrence, thereby adding prognostic granularity beyond standard histopathological examination alone. NN architectures further synergized clinical and imaging data, capturing nonlinear interactions between capsular invasion and neural risk [29]. Biological markers such as CEA may reflect intrinsic invasive potential, whereas radiomic features (eg, A9) represent its spatial manifestation. Notably, tumors with capsular invasion characterized by elevated CEA levels and disorganized textures (low A9 values) exhibited markedly increased NI rates, illustrating the model’s ability to decode multifactorial invasion pathways. The high performance of all models in the cross-label association analysis (eg, AUCs ≈0.80) is largely concordant with the strong pathological correlation between capsular invasion and NI identified by logistic regression (OR=25.25). This indicates that the models effectively captured the shared features underlying both invasion types, rather than demonstrating independent predictive power for NI on entirely unseen data.

Concerning clinical translation and distinction between model types, it is crucial to distinguish the intended clinical context of the different models developed in this study. The radiomic models (nomogram and RF based on features A1-A9), derived solely from preoperative CT, hold promise as preoperative decision-support tools. They could inform surgical planning, such as the extent of resection or the need for intraoperative nerve monitoring, based on noninvasive imaging. In contrast, the clinical biomarker-based models and the multimodal NN model integrate data (Gal-3 and CK19) that are only available postoperatively from resected tissue. Therefore, their primary utility lies in postoperative risk stratification. They may help pathologists and clinicians identify high-risk subsets among patients already diagnosed with capsular invasion, potentially predicting the likelihood of concurrent or subsequent NI, and thus informing adjuvant therapy strategies or the intensity of follow-up. This postoperative application remains valuable, as it could refine prognostic assessment beyond standard histology.

### Limitations and Future Directions

However, this study has several limitations. First, the multivariate logistic regression models (Tables S1 and S2 in Multimedia Appendix 3) are methodologically limited by circular reasoning. Using 1 postoperative pathological finding (eg, NI) to predict another (eg, capsular invasion) inflates the observed associations. The resultant astronomically high ORs with extremely wide CIs are classic signs of overfitting and multicollinearity within our dataset, rather than reliable measures of preoperative risk. These results should be interpreted as strong indicators of pathological co-occurrence, not as valid predictive effects.

Second, the single-center design, limited sample size (n=111), absence of external validation, and reliance on CT scanners from a single manufacturer may restrict generalizability and introduce selection bias.

Third, an additional limitation is the unconventional data usage design: the full dataset was used for both capsular invasion-modeling and cross-label association analysis without a separate held-out test set. Therefore, the reported AUCs for NI prediction are not directly comparable to studies using independent test sets. However, this design was necessary to maximize data use given the small sample size and to specifically test the cross-predictive value of capsular invasion–related features for NI, an exploratory goal that prioritizes verifying feature relevance across related clinical outcomes. Supplementary traditional 7:3 split validation confirmed consistent performance, mitigating concerns about overfitting to some extent (Multimedia Appendix 4).

Fourth is the sampling bias from single-slice analysis. Our NN model used only the single 2D slice with the largest cross-sectional tumor area. As capsular invasion predominantly occurs at the tumor boundaries or poles, this central slice may systematically exclude the imaging features most directly associated with capsular invasion, potentially limiting the model’s sensitivity to capture these specific patterns. Consequently, the “Image_Boundary” pattern identified by the network (Figure 2B) likely reflects the general sharpness or regularity of the tumor-parenchyma interface on the central slice, rather than specific imaging signs of focal capsular penetration at the tumor periphery.

Fifth, there is a limitation regarding the control group for qPCR analysis. The use of PBMCs from healthy individuals as a control does not yield biologically precise fold-change values for epithelial markers such as CK19, due to their near absence in PBMCs. In this study, the ≥2-fold threshold was used primarily as a sensitive, technical cutoff to categorize tumors into those with detectably high versus low or no expression of the target genes, facilitating subsequent clinical correlation analysis. This pragmatic approach should be distinguished from a biologically rigorous quantification of overexpression. Future studies should use paired adjacent normal thyroid tissue as the control to obtain accurate biological quantifications.

Sixth is the normalization-induced information leakage. The *z* score normalization of radiomic features was performed on the entire dataset before model development and cross-validation. This approach could theoretically allow information from validation samples (mean and SD) to leak into the training process, potentially inflating the model’s performance estimates. While we used cross-validation and an independent hold-out set to mitigate overfitting, this methodological choice may still contribute to an optimistic bias in the reported metrics. Future studies should consider performing normalization within each fold of the cross-validation loop to provide a more rigorous and unbiased evaluation of model generalizability.

Seventh is feature selection bias. In this study, the LASSO regression was applied to the entire dataset to select the 9 key radiomic features (A1-A9) before model development and cross-validation. Performing feature selection outside the cross-validation loop can introduce selection bias, as the selection process is informed by data that would later serve for validation. This may lead to overfitting and an optimistic estimation of the model’s generalizable performance. Consequently, the independence of the hold-out validation for the RF model is partially compromised. Future work should use a nested cross-validation framework, where feature selection is performed independently within each training fold, to obtain unbiased feature sets and more rigorous performance estimates.

Eighth is the potential misalignment from rigid registration. This study used rigid image registration to propagate arterial-phase ROIs to the venous-phase images for feature extraction (eg, feature A9, the venous-phase coefficient of variation). While efficient and commonly used, rigid registration assumes no tissue deformation between phases. Given that the thyroid is a soft organ susceptible to movement from swallowing or breathing, perfect alignment cannot be guaranteed. Any misalignment could cause venous-phase features to partially capture the texture of the tumor’s immediate periphery or surrounding tissue rather than the tumor itself. This may introduce noise or bias, particularly for texture-based features such as A9. Therefore, the high importance score of A9 in our model may partially reflect registration artifacts (such as edge effects at the ROI boundaries) rather than solely genuine intratumoral heterogeneity. Although we performed manual verification and adjustment of the propagated ROIs, minor residual misalignments may persist. Future studies could consider using deformable (nonrigid) registration techniques or independent manual delineation on venous-phase images to ensure more accurate feature localization and interpretation.

Ninth is the resolution loss due to image resizing for deep learning. In this study, to comply with the input requirements of the pretrained DenseNet121 network, the entire ROI was resized as a whole from its original resolution to 224×224 pixels. This means that no prior cropping to a tumor-specific bounding box was performed. For smaller thyroid tumors, this global down-sampling can substantially reduce the number of pixels representing the tumor tissue, leading to a loss of fine spatial detail and high-frequency texture information. This process may diminish the model’s ability to learn subtle textural patterns that are crucial for predicting capsular invasion, particularly in small lesions. Future studies could improve upon this by implementing a crop-to-bound-box step before resizing or by using NN architectures that support variable input sizes. Furthermore, the “learned image patterns” (eg, “Image_Heterogeneity” and “Image_Boundary”) identified through our interpretability analysis (SHAP) may therefore reflect macrolevel structures or artifacts introduced by the global down-sampling process, rather than solely the fine, tumor-specific textural details that are theoretically of interest. For instance, patterns interpreted as heterogeneity could be influenced by the interpolation artifacts during resizing or by the inclusion of nontumoral perinodular tissues within the ROI. While these patterns still contributed to the model’s predictive performance, their biological interpretability in relation to capsular invasion should be interpreted with this technical limitation in mind.

Tenth is the uncertainty in hold-out set performance. The performance of the NN model on the small independent hold-out set (n=33; AUC=0.827, 95% CI 0.652‐0.981) showed a point estimate higher than that from the full-sample cross-validation (AUC=0.775). The wide 95% CI reflects the substantial uncertainty inherent in evaluating a model with such a limited test sample. While the hold-out set was constructed using stratified sampling to maintain a representative distribution of capsular invasion status, the observed difference in point estimates may be attributable to the small sample size and its associated variability. Therefore, the hold-out performance should be interpreted with caution, and the model’s generalizability needs to be confirmed in larger, prospective cohorts.

Eleventh is the potential confounding in model comparison due to input differences. The radiomic RF model identified a venous-phase feature (A9) as highly predictive, while the NN was designed with arterial-phase-only inputs for simplicity and to mitigate overfitting. This design difference means that the lower AUC of the NN model (0.775) compared to the RF model (0.8102) may be partially attributable to the exclusion of venous-phase information rather than an inherent limitation of the deep learning architecture itself. Our study aimed to test whether a single phase (arterial) could provide a sufficient predictive signal for a deep learning model; however, this choice introduces a confounding factor when directly comparing performance across methodologies. Future studies could develop multimodal NN architectures incorporating both arterial and venous phases to enable a more equitable comparison and potentially enhance performance.

Future work will expand multicenter collaboration to include over 300 cases across diverse CT devices, incorporate data augmentation and explainable artificial intelligence methods, and establish standardized protocols to improve robustness. Most importantly, future models will focus on using exclusively preoperative variables to achieve genuine preoperative risk stratification.

### Conclusions

In conclusion, our exploratory analysis confirms a strong association between capsular invasion and NI in thyroid carcinoma. This study delineates complementary approaches for invasion risk assessment: (1) radiomic signatures from preoperative CT alone demonstrate potential for noninvasive, preoperative risk stratification; and (2) models incorporating postoperative tissue-derived biomarkers (eg, Gal-3 and CK19), including the multimodal model, provide a valuable framework for postoperative risk stratification. These postoperative tools could aid in identifying patients with high-risk pathological features, potentially guiding decisions on adjuvant therapy or surveillance intensity following surgery. The performance of the radiomic models lays the groundwork for future research toward fully preoperative predictive tools.

## Supplementary material

10.2196/77349 Multimedia Appendix 1 Detailed architecture of the neural network model for capsular invasion prediction.

10.2196/77349 Multimedia Appendix 2 Definitions and pathophysiological correlations of the 9 key radiomic features (A1-A9).

10.2196/77349 Multimedia Appendix 3 Detailed multivariate logistic regression analyses for capsular invasion (Table S1) and neural invasion (Table S2).

10.2196/77349 Multimedia Appendix 4 Hold-out validation results of the random forest model for neural invasion prediction.

## Abbreviations

AUC: area under the curve
CA125: carbohydrate antigen 125
CA199: carbohydrate antigen 199
CCSA-2: circulating cancer-specific antigen
CEA: carcinoembryonic antigen
CK19: cytokeratin 19
CT: computed tomography
Gal-3: galectin-3
GLCM: gray-level co-occurrence matrix
HBME-1: Hector Battifora Mesothelial Epitope-1
ICC: intraclass correlation coefficient
LASSO: least absolute shrinkage and selection operator
LDA: linear discriminant analysis
MAE: mean absolute error
NI: neural invasion
NN: neural network
OR: odds ratio
PBMC: peripheral blood mononuclear cell
PCA: principal component analysis
qPCR: quantitative polymerase chain reaction
RF: random forest
RGB: red, green, and blue
ROC: receiver operating characteristic
ROI: regions of interest
SHAP: Shapley Additive Explanations
XAI: explainable artificial intelligence
