# Supplementary material for: CT Radiomics–Based Machine Learning Model for Predicting Capsular and Neural Invasion in Thyroid Carcinoma: Diagnostic Accuracy Study
Source: JMIR Med Inform. 2026 Mar 12;14:e77349. doi: 10.2196/77349 (PMC12981638; doi:10.2196/77349)
Supplement: Multimedia Appendix 1 — Detailed architecture of the neural network model for capsular invasion prediction. [file medinform-v14-e77349-s001.docx]

| Module | Layer/Component | Detailed Specifications | Purpose (Linked to Manuscript Goals) |
| --- | --- | --- | --- |
| Input Layer | CT ROI Input | - Source: Arterial-phase CT ROIs (1.25 mm thin-section, GE Discovery CT)  - Preprocessing: Min-max scaled to [0,1], resized to 224×224 pixels (consistent with Section 1.2) | To comply with the fixed-size input requirement of the pre-trained DenseNet121 backbone. Note: This resizing step was exclusive to the deep learning model. Radiomic features (A1-A9) were extracted from original-resolution ROIs as stated in the main text (Section “Imaging feature extraction”) |
|  | Clinical Feature Input | - Variables: 7 clinical biomarkers (Gal-3, HBME-1, CK19, CEA, CA19-9, CA125, CCSA-2)  - Preprocessing: Min-max scaled to [0,1] (to align with CT feature range) | Normalize numerical differences between clinical indicators (e.g., CEA: ng/mL vs. CA199: U/mL) |
| Feature Fusion Layer | DenseNet121 Backbone | - Retained layers: First 10 convolutional blocks (freeze pre-trained ImageNet weights; avoid overfitting with small sample size)  - Output: 256-dimensional feature vector (via global average pooling) | Extract high-level spatial features from CT ROIs (e.g., texture, enhancement heterogeneity) linked to CI (Section 3 Discussion) |
|  | Clinical Feature Branch | - Structure: 1 fully connected layer (16 neurons, ReLU activation)  - Output: 16-dimensional feature vector | Compress high-dimensional clinical data to a compact space for balanced fusion with CT features |
|  | Feature Concatenation | - Operation: Merge 256D CT features + 16D clinical features  - Output: 272-dimensional fused vector | Integrate multimodal information (imaging + clinical) to capture complementary CI-related traits (Section 2.7) |
|  | Fused Feature Processing | - Structure: 1 fully connected layer (256 neurons, ReLU activation, no dropout)  - Output: 256-dimensional refined vector | Refine fused features to highlight key signals for CI prediction (training label) |
| Output Layer | Binary Classification Layer | - Activation: Sigmoid  - Output: 1-dimensional probability (0 = CI-negative, 1 = CI-positive; threshold = 0.5) | Match the study’s binary NI risk stratification goal (Section 2 Results) |
| Training Parameters | Optimizer | Adam optimizer, learning rate = 1×10⁻⁴ (fixed; tested in preliminary experiments to balance convergence speed and stability) | Ensure stable training on the 111-case cohort (avoid rapid divergence with small data) |
|  | Loss Function | Binary cross-entropy (adapted to binary CI/NI labels) | Minimize prediction error for CI (training) and NI (evaluation) binary outcomes |
|  | Training Controls | - Epochs: Max 50, early stopping triggered by 10 consecutive epochs of no validation loss reduction (Section 2.7)  - Batch size: 16  - Cross-validation: 5-fold (stratified by CI status) | Prevent overfitting (critical for small sample size) and ensure model generalizability to NI prediction |
