# Supplementary material for: CT Radiomics–Based Machine Learning Model for Predicting Capsular and Neural Invasion in Thyroid Carcinoma: Diagnostic Accuracy Study
Source: JMIR Med Inform. 2026 Mar 12;14:e77349. doi: 10.2196/77349 (PMC12981638; doi:10.2196/77349)
Supplement: Multimedia Appendix 2 — Definitions and pathophysiological correlations of the 9 key radiomic features (A1-A9). [file medinform-v14-e77349-s002.docx]

| Radiomic Feature | Feature Type (Pixel-Level Indicator) | CT Image Manifestation (Quantitative Description) | Potential Clinical/Pathological Correlation (Speculative) | Basis for Correlation |
| --- | --- | --- | --- | --- |
| A1 | Arterial-phase pixel intensity mean | Average gray value of all pixels in ROI; higher values indicate stronger contrast enhancement of the tumor | Associated with tumor vascular density and invasiveness: Higher A1 values may correspond to hypervascular tumors, which are more likely to invade the capsule (CI) and nerves (NI) | 1. Thyroid carcinoma with CI/NI often exhibits increased angiogenesis (Reference 21), leading to stronger arterial-phase enhancement; 2. In this study, A1 was significantly higher in CI-positive group (342.6 ± 41.8) than non-CI group (289.3 ± 37.5, P<0.001), and positively correlated with CA199 (r=0.38, P<0.01, a marker of tumor burden) |
| A2 | Arterial-phase pixel intensity standard deviation (SD) | Degree of dispersion of individual pixel gray values in ROI; higher values indicate uneven contrast enhancement within the tumor | Associated with intratumoral necrosis or cystic change: Higher A2 values may reflect focal necrosis (low-intensity pixels) mixed with viable tumor tissue (high-intensity pixels), a sign of aggressive tumor behavior | 1. Necrotic foci in thyroid carcinoma appear as low-density areas on arterial-phase CT, increasing pixel intensity SD (Reference 8); 2. A2 was significantly higher in NI-positive group (89.7 ± 12.3) than non-NI group (65.2 ± 10.8, P<0.001), and 76.5% of high-A2 cases (A2>80) had positive CK19 expression (a marker of poor differentiation) |
| A3 | Venous-phase pixel intensity 90th percentile | Gray value corresponding to the 90th percentile of all pixels in ROI; represents the upper limit of high-intensity pixels in the tumor | Associated with tumor capsule integrity: Lower A3 values may indicate capsule rupture, as contrast agent extravasation into surrounding tissues reduces the concentration of contrast in the tumor (lower high-intensity pixel proportion) | 1. Intact tumor capsules restrict contrast agent leakage, maintaining high venous-phase pixel intensity (high 90th percentile) (Reference 3); 2. A3 was significantly lower in CI-positive group (298.5 ± 35.1) than non-CI group (356.2 ± 40.3, P<0.001), and CI-negative cases had a 2.3-fold higher A3>320 rate than CI-positive cases |
| A4 | Arterial-phase pixel intensity skewness | Asymmetry of the pixel intensity distribution curve; positive values indicate a skewed distribution toward low-intensity pixels | Associated with intratumoral compositional heterogeneity: Positive skewness indicates a concentration of low-intensity pixels, potentially reflecting cystic degeneration or necrotic components within the tumor, which may influence local invasion patterns. | 1. Positive skewness reflects the presence of intratumoral cystic or necrotic components, as it indicates a concentration of low-intensity pixels with a tail of higher values from viable tissue., shifting the pixel intensity distribution to low values (positive skewness) (Reference 6); 2. A4 was positively correlated with A8 (capsule-related feature, r=0.42, P<0.01), and non-CI cases had a higher positive skewness rate (68.8%) than CI cases (27.0%, P<0.001) |
| A5 | Venous-phase pixel intensity range | Difference between the maximum and minimum pixel gray values in ROI; reflects the overall intensity variation of the tumor | Associated with tumor differentiation: Narrower A5 (smaller range) may correspond to well-differentiated tumors (uniform cell structure, consistent contrast enhancement); wider A5 may indicate poorly differentiated tumors (disorganized cells, uneven enhancement) | 1. Well-differentiated papillary thyroid carcinoma (PTC) shows uniform venous-phase enhancement (narrow pixel range) (Reference 17); 2. A5 was significantly wider in CK19-positive group (189.6 ± 25.4) than CK19-negative group (126.3 ± 21.7, P<0.001), and CK19 is a marker of PTC aggressiveness |
| A6 | Arterial-phase pixel intensity kurtosis | Peakedness of the pixel intensity distribution curve; higher values indicate a more concentrated distribution of pixel intensities | Associated with tumor cell density uniformity: Higher A6 values suggest densely packed, uniformly distributed tumor cells (consistent contrast uptake), which may increase the risk of perineural infiltration | 1. Densely packed tumor cells show homogeneous contrast enhancement, leading to a concentrated pixel intensity distribution (high kurtosis) (Reference 21); 2. A6 was significantly higher in NI-positive group (3.8 ± 0.7) than non-NI group (2.1 ± 0.5, P<0.001), and AUC of A6 for predicting NI was 0.72 (95%CI: 0.61–0.82) |
| A7 | Venous-phase pixel intensity median | Median gray value of all pixels in ROI; represents the "typical" enhancement intensity of the tumor | Associated with tumor proliferation activity: Higher A7 values may reflect active tumor cell metabolism and increased contrast agent uptake, correlating with elevated CEA levels (a proliferation-related marker) | 1. High metabolic activity in tumors enhances venous-phase contrast retention (high median pixel intensity) (Reference 19); 2. A7 was positively correlated with serum CEA (r=0.45, P<0.001), and CEA>35 ng/mL cases had a higher A7 (312.4 ± 38.2) than CEA≤35 ng/mL cases (265.7 ± 32.9, P<0.001) |
| A8 | Arterial-phase pixel intensity 10th percentile | Gray value corresponding to the 10th percentile of all pixels in ROI; represents the lower limit of low-intensity pixels in the tumor | Associated with capsule thickness and integrity: Higher A8 values (fewer extremely low-intensity pixels) may indicate a thick, intact capsule (no leakage of low-intensity interstitial fluid into the tumor) | 1. Intact capsules prevent low-intensity interstitial fluid from entering the tumor, increasing the 10th percentile of pixel intensity (Reference 3); 2. A8 was significantly higher in non-CI group (225.6 ± 28.3) than CI group (189.4 ± 25.7, P<0.001), and non-CI cases had a 0.3% rate of A8<200, compared to 34.9% in CI cases |
| A9 | Venous-phase pixel intensity coefficient of variation (CV) | Ratio of pixel intensity SD to mean; quantifies relative variation in enhancement within the tumor | Associated with the risk of neural invasion (NI): Higher A9 values indicate more heterogeneous venous-phase enhancement, which may reflect tumor infiltration into perineural spaces (uneven contrast uptake in invasive foci) | 1. Tumors invading perineural spaces show heterogeneous enhancement due to mixed tumor cells and nerve tissue, increasing CV (Reference 8); 2. A9 was the most important pixel-level feature in the radiomic-based random forest (RF) model (importance score=89), and NI-positive cases had a higher A9 (0.32 ± 0.06) than non-NI cases (0.18 ± 0.04, P<0.001) |

Technical Note: The radiomic features A1-A9 described in this table were extracted from the original-resolution CT ROIs (without resizing) to preserve the physical scale and meaning of the texture metrics. The resizing of ROIs to 224×224 pixels was a separate preprocessing step applied only for input into the DenseNet121-based neural network model.
