# Supplementary material for: CT Radiomics–Based Machine Learning Model for Predicting Capsular and Neural Invasion in Thyroid Carcinoma: Diagnostic Accuracy Study
Source: JMIR Med Inform. 2026 Mar 12;14:e77349. doi: 10.2196/77349 (PMC12981638; doi:10.2196/77349)
Supplement: Multimedia Appendix 3 — Detailed multivariate logistic regression analyses for capsular invasion (Table S1) and neural invasion (Table S2). [file medinform-v14-e77349-s003.docx]

**Table S1.** Multivariate binary logistic regression analysis for capsular invasion.

|  | B | SE | W | *P* | OR | 95% CI | |
| --- | --- | --- | --- | --- | --- | --- | --- |
|  |  |  |  |  |  | Lower | Upper |
| Gal-3 | 1.65 | 0.895 | 3.401 | 0.065 | 5.208 | 0.902 | 30.083 |
| CK19 | 4.102 | 1.171 | 12.269 | <0.001 | 60.491 | 6.092 | 600.654 |
| CEA | 0.244 | 0.092 | 7.064 | 0.008 | 1.276 | 1.066 | 1.528 |
| CCSA-2 | 0.123 | 0.076 | 2.603 | 0.107 | 1.131 | 0.974 | 1.313 |
| CA199 | 0.14 | 0.043 | 10.359 | 0.001 | 1.15 | 1.056 | 1.252 |
| CA125 | 0.092 | 0.036 | 6.336 | 0.012 | 1.096 | 1.02 | 1.177 |
| NI | 3.884 | 1.322 | 8.63 | 0.003 | 48.641 | 3.643 | 649.434 |
| Constant | -32.362 | 7.395 | 19.154 | <0.001 |  |  |  |

**Table S2.** Multivariate binary logistic regression analysis for neural invasion.

|  | B | SE | *W* | *P* | OR | 95% CI | |
| --- | --- | --- | --- | --- | --- | --- | --- |
|  |  |  |  |  |  | Lower | Upper |
| Gal-3 | -0.742 | 0.528 | 1.974 | 0.16 | 0.476 | 0.169 | 1.34 |
| CK19 | 0.156 | 0.975 | 0.025 | 0.873 | 1.168 | 0.173 | 7.901 |
| CEA | -0.074 | 0.06 | 1.514 | 0.219 | 0.929 | 0.825 | 1.045 |
| CCSA-2 | -0.032 | 0.053 | 0.378 | 0.539 | 0.968 | 0.873 | 1.074 |
| CA199 | 0.001 | 0.024 | 0.001 | 0.971 | 1.001 | 0.955 | 1.049 |
| CA125 | 0.002 | 0.017 | 0.018 | 0.892 | 1.002 | 0.97 | 1.035 |
| CI | 3.229 | 0.946 | 11.649 | 0.001 | 25.25 | 3.954 | 161.26 |
| Constant | 0.252 | 3.276 | 0.006 | 0.939 | 1.286 |  |  |
