# Supplementary material for: CT Radiomics–Based Machine Learning Model for Predicting Capsular and Neural Invasion in Thyroid Carcinoma: Diagnostic Accuracy Study
Source: JMIR Med Inform. 2026 Mar 12;14:e77349. doi: 10.2196/77349 (PMC12981638; doi:10.2196/77349)

7:3 Hold-Out Validation Results

To address methodological concerns, a 7:3 hold-out validation was conducted. The random forest model, trained on capsular invasion features, achieved an AUC of 0.827 (95% CI: 0.652-0.981) for neural invasion prediction on the independent test set (training: n=78; test: n=33).

**Figure S1.** ROC curve of the 7:3 hold-out validation for neural invasion prediction (AUC = 0.827, 95% CI: 0.652-0.981).


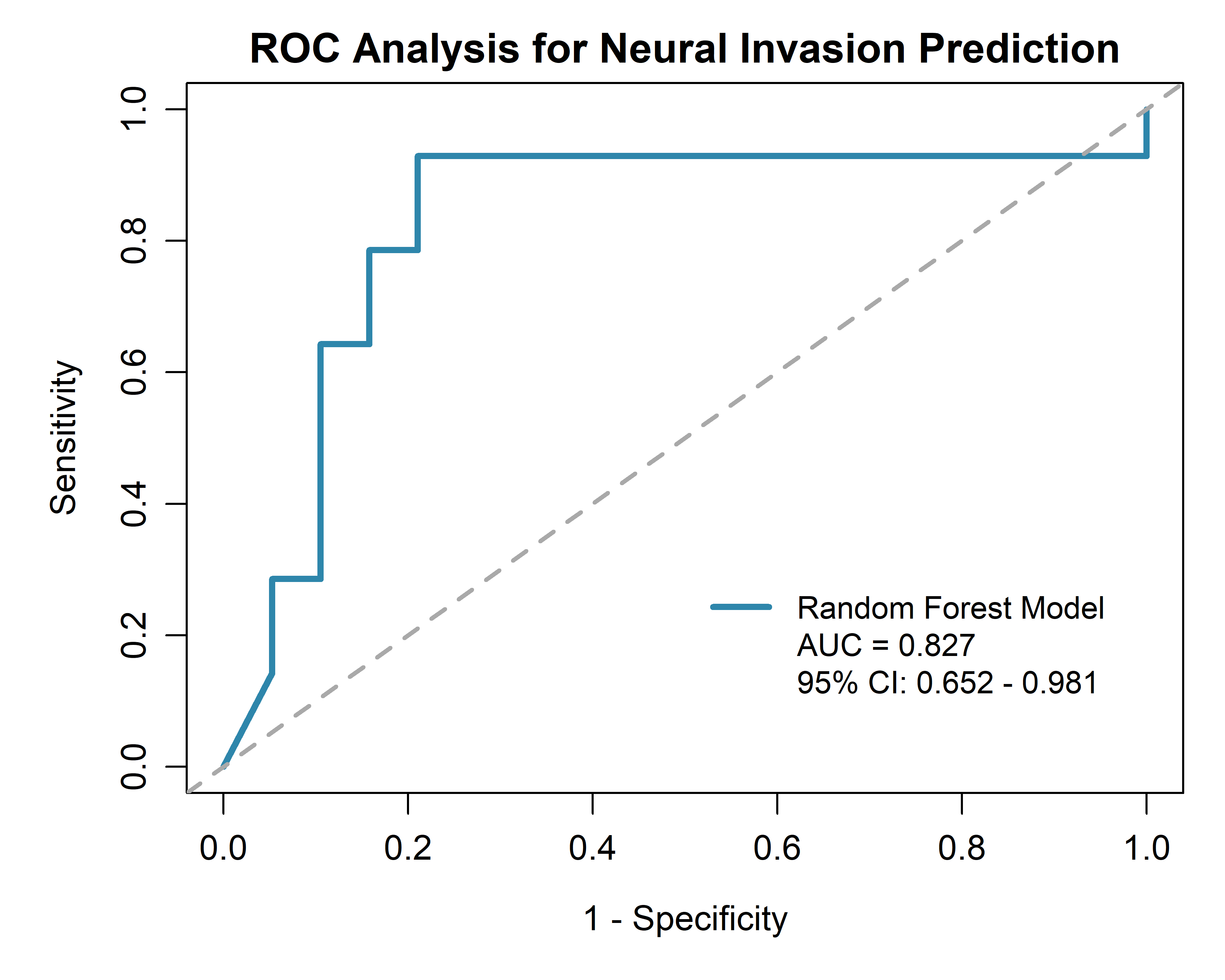

Supplement: Multimedia Appendix 4 — Hold-out validation results of the random forest model for neural invasion prediction. [file medinform-v14-e77349-s004.doc]
